# Are oxidized low-density lipoprotein and C-reactive protein markers of atherosclerosis in nephrotic children?

**DOI:** 10.1007/s11845-014-1170-8

**Published:** 2014-07-24

**Authors:** A. Rybi-Szumińska, A. Wasilewska, J. Michaluk-Skutnik, B. Osipiuk-Remża, R. Fiłonowicz, M. Zając

**Affiliations:** Department of Pediatrics and Nephrology, Medical University of Białystok, 17 Waszyngton Street, 15-274 Bialystok, Poland

**Keywords:** Idiopathic nephrotic syndrome, Oxidative stress, Atherosclerosis, OxLDL, hs-CRP

## Abstract

**Background:**

Lipid disorders are known to be linked to disturbance in oxidative reactions and play an important role in the progression and complications of idiopathic nephrotic syndrome (INS).

**Aims:**

The aim of this study was to assess oxidized low-density lipoprotein (oxLDL), high-sensitive C-reactive protein (hs-CRP) serum concentrations and other parameters of lipid metabolism in children with INS during relapse and remission of proteinuria.

**Methods:**

The examination was performed on 23 children and adolescents diagnosed with INS. Reference group consisted of 22 participants. The study was carried out twice: in the relapse of INS (A) and in remission of proteinuria during glucocorticoid treatment (B).

**Results:**

OxLDL was higher in INS patients, in both examinations when compared with reference participants. hs-CRP showed no differences between nephrotic and healthy children. We found higher concentration of oxLDL in children, who where frequent relapsers. Cholesterol, triglycerides/high density lipoprotein cholesterol and platelets were higher in INS patients (both A and B) in comparison with healthy children.

**Conclusions:**

We observed presence of pro-atherogenic lipid profile in INS. Elevation of oxLDL may reflect increased oxidative stress and higher risk of atherosclerosis in INS, therefore it seems to be relevant to find patients of risk of atherosclerosis to consider lipid lowering treatment with antioxidants.

## Introduction

Oxidative stress (OS), defined as disturbance in the reactive oxygen species (ROS) and antioxidant balance, can result either from low levels of antioxidants and/or from an. increased production of ROS. Oxidative stress has been previously demonstrated in various kidney disorders such as glomerulonephritis [1] and acute renal injury [2] or diabetic nephropathy [3]. Experimental studies showed that ROS also play an important role in the pathogenesis of experimental puromycin aminonucleoside induced nephrosis [4]. It has been postulated that childhood idiopathic nephrotic syndrome (INS) is associated with OS due to increased levels of ROS and decreased levels of antioxidants [5]. Mishra et al. [2] reported evidence of OS and impaired antioxidant defense during acute INS. Previous studies have demonstrated presence of oxidative stress in children with idiopathic nephrotic syndrome, but there is paucity of data regarding its status among different sub-groups of patients [6–8]. An important advance in understanding the pathogenesis of INS was the observation that oxygen-free radicals are possible mediators of injury in experimental nephrosis in rats. The similarity of that animal model to human minimal change nephrotic syndrome (MCNS) provokes the idea that free-radical mediated injury could play a role in the pathogenesis of that disorder [9]. This is probably a consequence of an imbalance between oxidant and antioxidant activity in vivo [10]. Furthermore, dyslipidemia in INS is also known to be linked to oxidative reactions and atherosclerosis [11]. Patients with INS have elevated concentrations of total cholesterol (TC), LDL cholesterol (LDL-C), and triglycerides (TG), whereas, HDL cholesterol (HDL-C) has variously been reported to be increased, decreased, or normal [5]. Lipoproteins: Lp(a), ox-Lp(a) and Lp(a)-IC levels were increased in INS children, which may play an important role in the processes of atherosclerosis [12]. These abnormalities suggest that nephrotic patients have higher risk of developing atherosclerosis.

In the last three decades, a large volume of studies have established that oxidized low-density lipoprotein (oxLDL) is a useful marker of cardiovascular diseases (CVDs) [12, 13]. The measurement of oxLDL correlates with the presence of CVDs and indicates that oxLDL is a potential prognostic indicator for future health events. OxLDL is known to stimulate macrophages to induce foam cell formation and inflammatory responses. We read with a great interest paper of Zhang et al. [14], who found a positive significant correlation between oxLDL and C-reactive protein (CRP) in patients with acute coronary syndrome (ACS). This study brings new insights that oxLDL and CRP may play a direct role in promoting the inflammatory component of atherosclerosis.

The aim of this study was to assess oxLDL and hs-CRP serum concentrations in children with INS.

## Methods

Written informed consent was obtained from parents and all participants older than 16 years old. The protocol was approved by the Bioethics Committee of the Medical University of Bialystok in accordance with the Declaration of Helsinki.

The examination was performed on a group of 23 children and adolescents diagnosed with INS according to the definition of the International Society of Kidney Disease in Children [15], treated in the Department of Pediatrics and Nephrology, in years 2011–2012. All nephrotic patients who fulfilled the inclusion criteria were included in the study. Inclusion criteria were: steroid-sensitive nephrotic syndrome (SSNS), age at the time of the study (1.8–18 years), normal blood pressure during examination, normal GFR (>90 ml/min/1.73 m^2^), according to Schwartz formula, absence of clinical and laboratory findings of a systemic disease, accomplishing the criteria of a relapse of NS. Exclusion criteria: signs of an acute infection (body temperature >37 °C), presence of other chronic diseases, immunosuppressive treatment other than glucocorticoids (GCS) therapy.

The testing was performed twice: A, in relapse of NS, before starting GCS therapy and B, in remission of proteinuria (at least 3 days without proteinuria in urinalysis), during GCS treatment. All participants with INS were treated with the standard GCS initial therapy—daily dosage of prednisone 60 mg/m^2^ body surface area for 4 weeks, followed by 40 mg/m^2^ given on alternate days, followed by various tapering on alternate days. Relapses were treated with daily prednisone 60 mg/m^2^ until remission was achieved, followed by 40 mg/m^2^ on alternate days.

Reference group consisted of 22 healthy children and adolescents (M: 11, F: 11), aged 0.7–18 years, diagnosed in our Department because of monosymptomatic nocturnal enuresis. They did not have any signs of acute or chronic disease and did not take any drugs. Some participants were also healthy children of the personnel working in the Department. Health status was determined through the subjects’ medical history, parental report and routine laboratory tests.

All samples were collected after 12 h of night fasting. Blood samples were drawn to measure serum concentrations of: oxLDL, hs-CRP, albumin, total cholesterol, LDL cholesterol, HDL cholesterol, triglycerides and platelets count. For the purpose of the assay 650 μl of sera was obtained from residual blood sample. Serum samples were stored at −80 °C until assayed.

Urinalysis was performed to assess protein level in the morning sample. Both oxLDL and hs-CRP serum concentrations were determined using ELISA method, according to the instruction given by the manufacturer (oxLDL ELISA Kit, Immunodiagnostik AG, hs-CRP—LDN Labor Diagnostika Nord GmbH & Co.KG). The detection limit of oxLDL and hs-CRP were 4.13 and 10 ng/ml, respectively. The intra and inter-assay coefficients of variation (CV) for both of them were: 3.9 and 11 % for oxLDL and 15.2 and 9.9 % for hs-CRP.

The data were analyzed using the Statistica 10.0 package. Adequacy of parameters to normal distribution was tested by using Shapiro–Wilk Test. The Mann–Whitney *U* test was used to compare independent data when the variables did not follow a normal distribution. Correlations between clinical and laboratory parameters were tested by Spearman correlation coefficients. Chi-square test was used for categorical variables. Multiple regression model was created for the oxLDL as a dependent variable. Value of *p* < 0.05 was considered statistically significant.

## Results

Table 1 shows characteristics of examined subjects in relapse of INS (A), after resolution of proteinuria (B) and reference group (R). Children with INS were in similar age as reference participants. The proportion of male was greater in nephrotic children what is consistent with the demographics of childhood INS [16].

**Table 1:** Clinical and metabolic characteristics of subjects entered the study and the reference group

Variable	A (relapse with proteinuria)	B (relapse without proteinuria)	R (reference group)	*p* *AvB **AvR
Age (years)	4 (1.8–18)	4 (1.8–18)	5.75 (0.7–18)	*NS **NS
Sex (M/F) (%M)	15/8 (65.2 %)	15/8 (65.2 %)	11/11 (50 %)	*NS **NS
Albumin (g/l)	1.82 (0.98–3.1)	2.79 (1.89–3.62)	4.83 (3.9–4.83)	*<0.01 **<0.01
Cholesterol (mg/dl)	376 (185–724)	352 (166–495)	170 (106–203)	*NS **<0.01
HDL-C (mg/dl)	77 (32–116)	76.5 (26–314)	55 (26–77)	*NS **<0.05
Triglycerides (mg/dl)	259 (69–377)	205.5 (123–351)	68.5 (53–115)	*NS **<0.01
TG/HDL ratio	3.99 (0.76–8.32)	2.5 (0.68–7.89)	1.48 (0.69–3.1)	*NS **<0.01
Patelets count (×10^3^/mm^3^)	390 (199–920)	421 (270–780)	220 (149–456)	*NS ** < 0.01
hs-CRP (mg/l)	2.97 (1.08–84.67)	3.76 (1.16–98.5)	3.01 (1.16–8.22)	*NS **NS
OxLDL (ng/ml)	357.19 (40–2,500)	320.8 (23.01–2,500)	85.5 (40–572.91)	*NS **<0.05

All values are median and range, unless otherwise noted

*HDL-C* high density lipoprotein cholesterol, *TG/HDL* triglicerides/high density lipoprotein cholesterol, *hs-CRP* high sensitive C-reactive protein, *OxLDL* oxidized low-density lipoprotein, *p* level of statistical significance, *NS* without statistical significance

We found significantly higher serum concentration of TC, LDL-C and TG (*p* < 0.01) as well as HDL-C (*p* < 0.05) in nephrotic children in comparison with reference subjects. In INS patients we did not observe any significant decrease of serum levels of these biochemical parameters in examination B.

**Fig. 1:**
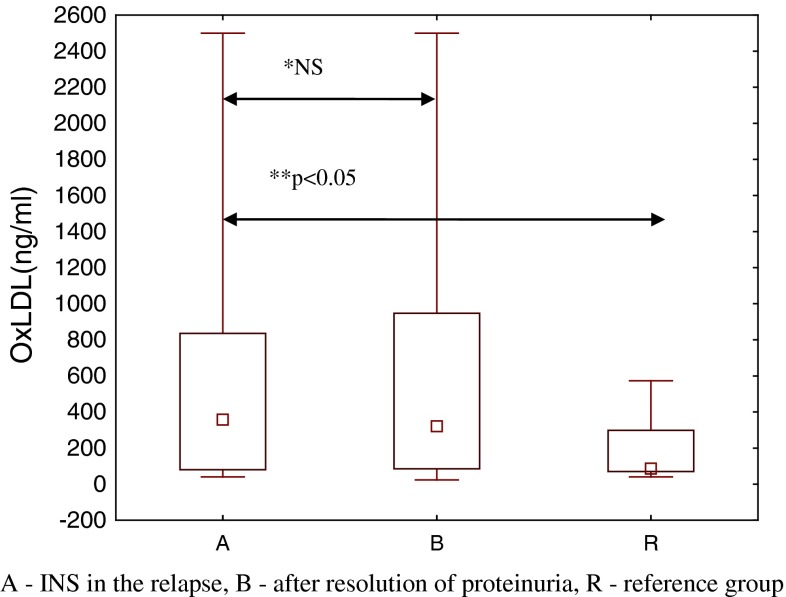
Comparison of oxLDL serum concentrations between studied groups

Nephrotic patients (group A and B) had also much higher platelets count (PLT) in comparison with reference group (*p* < 0.01). Obviously albumin concentration was much lower in INS participants before treatment (group A) in comparison with both INS patients after resolution of proteinuria (*p* < 0.01) and reference children (*p* < 0.01). OxLDL concentration was significantly higher in children with INS, both group A and B in comparison with reference group (*p* < 0.05) Fig. 1, while serum hs-CRP showed no statistically significant differences between children with INS and reference group. Atherogenic index—TG/HDL-C—was significantly higher in INS patients, both before and during GCS treatment (*p* < 0.01). We also analyzed oxLDL concentration in nephrotic patients in relation to the number of relapses. We found significantly higher concentration of oxLDL in children who where frequent relapsers (*p* < 0.01).

We assessed correlations between examined markers (oxLDL, hs-CRP) and other biochemical parameters in all examined children.

There were positive correlations between: oxLDL and PLT count (*r* = 0.26, *p* < 0.05), oxLDL and TG (*r* = 0.36, *p* < 0.05), oxLDL and TG/HDL ratio (*r* = 0.53, *p* < 0.05) and oxLDL and hs-CRP (*r* = 0.31, *p* < 0.05). The factors that were found to have significant correlation with oxLDL serum concentration in the single regression analyzes were used as explanatory variables to create the multiple regression model. To reduce the impact of multicollinearity we removed some correlated variables. In the model, remaining parameters (hs-CRP, PLT count and TG/HDL ratio) accounted for more than 33 % of the variations in oxLDL level (*r* = 0.51, *p* < 0.01); Table 2.

**Table 2:** Multiple linear regression analysis of oxLDL

	Coefficient	Standard error	95 % Confidence interval	*p* value
hs-CRP	4.89	6.49	−8.31–18.09	0.46
PLT	−0.38	0.48	−1.35–0.59	0.43
TG/HDL	126.56	55.31	14.03–239.08	0.03

*hs-CRP* high sensitive C-reactive protein, *PLT* platelets count, *TG/HDL* triglicerides/high density lipoprotein cholesterol

We also observed positive correlation between hs-CRP and TG/HDL ratio (*r* = 0.37, *p* < 0.05) and negative correlation between hs-CRP and HDL-C (*r* = −0.38, *p* < 0.05). Neither oxLDL nor hs-CRP correlated with serum albumin or proteinuria.

## Discussion

In this cross-sectional study we evaluated serum concentrations of oxLDL and hs-CRP in patients with INS and assessed correlation between these parameters and other markers of atherosclerosis. The lipids profile of INS patients revealed a typical pattern with elevated serum concentration of TC, LDL-C, TG. We also found significant hypoalbuminemia in INS children. There were significantly higher oxLDL values in nephrotic children in comparison to healthy subjects. We observed significantly higher concentration of oxLDL in children who where frequent relapsers irrespectively of a current relapse treatment.

Laboratory, clinical and pathological findings of the last decades proved that atherosclerosis is a process beginning in early childhood and progressing into adulthood [17]. Its main result is a great risk of coronary heart disease. It is obvious that high serum cholesterol level in childhood increases the risk of developing atherosclerosis in adults. Young patients with INS are more endangered with cardiovascular disease because of hyper- and dyslipidemia. However, it is considered that the risk of fast progression of atherosclerosis in nephrotic patients with good response to GCS therapy seems to be minimal, because the state of hypercholesterolemia is of short duration and intermittent. The most endangered group of patients with NS are those with unremitting proteinuria, frequent relapses and/or poor response to GCS and long standing hypoalbuminemia [18].

Hyper- and dyslipidemia are one of the most typical features of NS, with elevated serum levels of TC, LDL-C, TG and usually decreased HDL-C. This pro-atherogenic profile of lipids existing in NS is, in connection with lipid peroxidation, responsible for initial glomerular injury as well [19]. An escalation of lipid peroxidation and the release of free radicals (ROS) being strong oxidants result in glomerular injury and proteinuria [20]. They disrupt the integrity of membrane filtration barrier and impair capillary walls [21].

Observations of the last few years suggested that oxLDL may play an important role not only in the formation of atherosclerotic lesions but also in the progression of glomerulosclerosis [12, 22]_._ OxLDL enhances the expression of proinflammatory genes and recruits monocytes into vessel walls [23]. It acts through inflammatory signaling pathway CD40/CD40L and increases expression of matrix metalloproteinases genes (MMP-1 and-3) in endothelium [1, 2]. Its cytotoxic activity towards endothelium is related to production of ROS and impairment of nitric oxidase synthase gene as well [24]. Oxidative stress and increased serum concentration of oxLDL are strong mitogens for smooth muscles [25]. What is more, oxLDL particles are taken up by macrophages that become foam cells—forming fatty plaques in the tunica intima in arteries.

OxLDL serum concentration was higher in INS children in comparison to reference group. Nagla et al. who examined oxidative modification of LDL in 40 children with steroid sensitive nephrotic syndrome, also found significantly higher serum levels of oxLDL in this group of children in comparison with healthy volunteers, with especially high values in relapse group [12].

We found significantly higher concentration of oxLDL in children being frequent relapsers (more than 2 relapses of NS), irrespectively of presence of proteinuria. It seems that presence of proteinuria during relapse is not the main factor of increased risk of developing atherosclerosis. Also lack of correlation between oxLDL and proteinuria or serum albumin may confirm this thesis. Results of our investigation suggest that serum oxLDL concentration may be especially increased in INS patients with multiple relapses. Because of frequent, long standing proteinuria and dyslipidemia connected with toxic disturbances in oxidative status, they have relatively higher risk of atherosclerosis and elevated oxLDL may reflect this situation. This observation is consistent with Lechner et al. [26] who showed that long standing and frequently relapsing nephrotic patients are those, who are more endangered with an increased risk of cardiovascular complications. In children with minimal change nephrotic syndrome who are steroid sensitive, the risk of premature atherosclerosis is quite low. Glicocoticosteroids are not likely to have any influence on oxLDL values—it was similar during relapse, before treatment and similarly in the same children after resolution of proteinuria. This is consistent with observation of Nagla et al. [12] who did not find any normalization of studied markers despite of use of GCS until remission of proteinuria. What is more, there are some experimental findings suggesting that GCS impairs antioxidant status and stimulates production of ROS [27]. On the other hand the study of Kawamura et al. [28] emphasized therapeutic role of GCS therapy in stimulation of activity of antioxidant factors.

According to studies of last decades, inflammation is another process playing a pivotal role in the development and progression of atherosclerosis and cardiovascular disease [29]. Its main marker is an acute phase protein—C-reactive protein (CRP). It was shown that even slightly increased CRP serum concentration—detected as high-sensitive CRP (hs-CRP)—is associated with increased risk of atherothrombotic events in healthy participants. CRP is able to bind directly to LDL-C particles, especially oxidized (oxLDL) and is deposited in atheromathous plaques [30]. Moreover, its proinflammatory properties further stimulate progression of atheroma. However in our study hs-CRP values did not differ between NS patients and healthy volunteers. This observation is consistent with Ece et al. [11] who tested anti-oxidant status in relation to pro-inflammatory cytokines in INS children. Perhaps the proinflammatory and proatherogenic role of hs-CRP is not as important as escalating oxidative stress responsible for lipids peroxidation and higher oxLDL values in frequently relapsing nephrotic children.

Although we have not found any difference between hs-CRP concentration in nephrotic and healthy children, we noticed positive correlation between hs-CRP and approved atherosclerosis indicator TG/HDL-C ratio in children with relapse of INS after resolution of proteinuria. In studies of Protasio Lemos da Luz et al. elevation in the TG/HDL-C ratio is the most powerful predictor of coronary heart disease irrespectively of other lipid serum markers [31].We also noticed positive correlation between oxLDL and two approved atherosclerosis indicators hs-CRP and TG/HDL-C ratio. Also Zhang et al. [14] confirmed positive correlation between oxLDL and CRP in patients with acute coronary syndrome.

Our observation showed significantly higher concentration of TG/HDL-C ratio in INS patients and elevated PLT count in this group of children. Increased PLT count is another factor of development of atherothrombosis. This may confirm higher risk of atherosclerotic complications in the course of INS especially in those children who are steroid resistant and frequent relapsers and cannot achieve long-lasting remission with normalization of dyslipidemia.

In conclusion, the results of this study confirm presence of typical pro-atherogenic lipid profile in children with INS. Elevation in oxLDL concentration in frequent relapsers may reflect increased oxidative stress and higher risk of atherosclerosis in these group of patients. GCS therapy did not influence on concentration of pro-atherogenic biochemical bionicles—therefore it seems to be relevant to find patients of high risk of atherosclerosis and CVD to consider lipid lowering treatment combined with antioxidants. This study has some limitations. The main is relatively small sample size. It seems to be relevant to perform the examination on a larger group of patients. In the future, participants should be sex matched more carefully. It might be interesting to enroll patients in remission of INS, without GCS treatment. This could enable to draw more obligatory conclusions.
